# Knowledge, attitudes and practices of primary healthcare professionals to female genital mutilation in Valencia, Spain: are we ready for this challenge?

**DOI:** 10.1186/s12913-018-3396-z

**Published:** 2018-07-24

**Authors:** Alba González-Timoneda, Vicente Ruiz Ros, Marta González-Timoneda, Antonio Cano Sánchez

**Affiliations:** 1 Faculty of Nursing and Chiropody, University of Valencia, Calle Jaume Roig, s/n, 46010 Valencia, Spain; 2 University of Valencia, Valencia, Spain; 3 Obstetrics and Gynaecology Service, Hospital Clínico Universitario of Valencia, Av. de Blasco Ibáñez, 17,46010 Valencia, Spain; 4 Faculty of Medicine and Dentistry, Department of Obstetrics, Gyneacology and Peadiatrics, University of Valencia, Av. de Blasco Ibáñez, 15,46010 Valencia, Spain

**Keywords:** Female genital mutilation, KAP study, Women’s health, Professional practice, Primary health care

## Abstract

**Background:**

The practice of Female Genital Mutilation (FGM) is a deeply-rooted tradition in 30 Sub-Saharan and Middle-East countries which affects approximately 200 million women and girls worldwide. The practice leads to devastating consequences on the health and quality of life of women and girls in both the short and long term. Globalizing processes and migration flows have recorded cases of this practice worldwide representing for healthcare professionals an emerging challenge on how to approach their healthcare in a transcultural, ethical and respectful way. No survey to assess knowledge, attitudes and practices on FGM among primary healthcare professionals has been conducted in the Valencian region of Spain to date.

**Methods:**

The main purpose of this study is to assess the perceptions, knowledge, practices and attitudes of the primary healthcare professionals in relation to FGM in the Clínic-Malvarrosa healthcare area of Valencia. A cross-sectional descriptive study was conducted based on a self-administered questionnaire to general practitioners, paediatricians, nurses, midwives, gynaecologists, social workers and others.

**Results:**

A total of 321 professionals answered the questionnaire. Less than 5% of professionals answered that they had ever found a case of FGM during their professional practice and 21.8% answered that they had ever worked with population at risk of FGM. Almost 15% of professionals answered that they had received training on FGM but of those who had received training, only 22.7% correctly identified the typology of FGM and less than 5% correctly identified the geographical area. Only 6.9% of the respondents admitted to know some protocol of action, being midwives, paediatricians and social workers the most aware professionals of such protocols.

**Conclusion:**

This study demonstrates that FGM is a problem present in the population attending primary healthcare services in Valencia. However, the professionals showed a profound lack of knowledge around concept, typology, countries of prevalence of FGM and existent protocols of action. It is healthcare professional duty to recognize this situation and to follow the right protocols of action, refer these women and their families to the most appropriate services and professionals that fit their needs, ensuring a multidisciplinary, positive and transcultural care for these families.

**Electronic supplementary material:**

The online version of this article (10.1186/s12913-018-3396-z) contains supplementary material, which is available to authorized users.

## Background

The World Health Organization (WHO) published a report in April of 1997 in collaboration with UNICEF and UNFPA to define the term of female genital mutilation, also called “ablation” or “female circumcision”. It states that Female Genital Mutilation (FGM) comprises all procedures that involve partial or total removal of the female external genitalia, or other injury to the female genital organs for non-medical reasons [1]. The WHO/UNICEF/UNFRA Joint Statement classified female genital mutilation into four types as shown in Table 1.

**Table 1:** Types of Female Genital Mutilation

Type	Description
I	Clitoridectomy This is the partial or total removal of the clitoris (a small, sensitive and erectile part of the female genitals), and in very rare cases, only the prepuce (the fold of skin surrounding the clitoris).
II	Excision This is the partial or total removal of the clitoris and the labia minora with or without excision of the labia majora.
III	Infibulation This is the narrowing of the vaginal opening through the creation of a covering seal. The seal is formed by cutting and repositioning the labia minora or labia majora, sometimes through stitching with or without removal of the clitoris.
IV	Others This includes all other harmful procedures to the female genitalia for non-medical purposes, e.g. pricking, piercing, incising, scraping and cauterizing the genital area.

Source: Classification of the World Health Organization, 1995

According to data supplied by UNICEF in 2016, this practice affects approximately 200 million women and girls worldwide. FGM is carried out in 30 countries, mainly in sub-Saharan Africa, the Middle East (Egypt, Oman, Yemen and The Arab Emirates) and in some countries of Asia (India, Indonesia, Malaysia, Pakistan and Sri Lanka) with wide variations in prevalence. There are also some cases reported in indigenous areas from Latin America (Brazil, Colombia, Mexico and Peru). However, FGM is not practiced in all African countries, nor is it practiced by all ethnic groups within the same country [2, 3].

Due to globalizing processes and migration flows, some cases of this practice have been recorded worldwide, essentially in North America, Australia and Europe, where some migrant communities originally from countries with a high prevalence of FGM carry out this practice because of their desire of maintaining their traditions and to strengthen their cultural identities [3, 4]. Therefore, they practice the FGM in their country of residence o even in their home country (including bordering countries) when they come back to their cities on holidays. The WHO estimates that in Europe around 500,000 women and young females have experienced FGM and it considers that 180,000 young girls are at risk of being injured every year, even though these figures could be underestimated, since undocumented or second generation immigrants are not counted [5].

FGM leads to devastating consequences on the health and quality of life of women and girls in both the short and long term, yet this sort of ancestral ritual is deeply rooted in the cultural system of some communities and continues to be carried out despite it does not confer any health benefit [6, 7]. The reasons for this practice are very varied and complex. They are basically related to tradition, inequalities of power, preservation of virginity-chastity, social acceptance (mainly towards marriage), hygiene, increased male sexual pleasure, family honour, group membership and religion (misconception of being a religious requirement) [8]. It is a continuation of the history of social control of female sexuality and a form of gender inequality and discrimination against women.

FGM constitutes a fundamental violation of human rights, of women and girls as described in many international conventions. It is a discriminatory activity and violates the right to equal opportunities in life; the right to the highest level of health; the right to freedom from all forms of physical and mental violence, injury or abuse; the right to protection against all forms of traditional practices detrimental to health; the right to make reproductive decisions free from discrimination, coercion and violence; the right to freedom from prejudice and all other practices that are based on the idea of inferiority or superiority or of the gender or stereotyped roles of men and women [3, 4, 9, 10].

Although in Spain some cases of FGM were detected in Catalonia in 1993 and later in Palma de Mallorca in 1996, there is no reliable evidence that more female genital mutilations have been carried out in Spain. However, there have been cases of mutilated immigrants, especially in Catalonia and Andalusia [11–13]. Nevertheless, there is no national statistical database by health system of female genital mutilated residents in Spain, without prejudice to the data collected at the regional level in several Protocols of Action in the presence of FGM (Government of Catalonia, Government of Aragon, Government of Nav6arre and recently the Government of the Valencian Community) [12, 14–17].

The population residing in Spain from the countries where FGM is practiced originates mainly from Nigeria, Senegal, Gambia and Guinea and this population resides mainly in the Autonomous Communities of Catalonia, Madrid, Andalusia, the Valencian Community and the Basque Country [12, 13]. Over the decade of the 1990s and the early years of the 2000s there was a massive expansion of population from countries with prevalence of FGM in Spain, reaching historically high levels in 2008 before decreasing slowly.

According to the information included in the 2017 report by Kaplan et al., there are in Spain 69,086 women from countries in which FGM is practiced, representing a 5.2% increase since 2012. Of these, 18,396 are girls aged 0 to 14 years old, a group which has reduced slightly by 0.35% in the past four years. When focusing on data for each province, Barcelona houses the majority of sub-Saharan migrants, with 12,360 women and 3569 girls aged 0 to 14. In the second place is Madrid, followed by Girona, with 4538 women and 1409 girls, and Valencia with 3276 women and 950 girls aged 0 to 14. The Malian and Ghanean population are the ones who most have increased their female population (20% increased since 2012) [13].

In the Valencian Community (including the regions of Castellón, Valencia and Alicante), 19,934 people come from countries where FGM is practiced, 5429 of which are women and girls, 1268 are under 15 years of age and 4161 are over 15 years of age, according to data from the 2016 Population Information System [13].

In Spain, FGM in any of its manifestations is a crime of injury and is punishable by imprisonment of between 6 and 12 years, as provided in Section 149.2 of Organic Law 10/1995 of 23 November, of the Criminal Code (modified by Organic Law 11/2003).^1^ In addition, Spanish jurisdiction is also competent to prosecute FGM not only in Spanish territory but also in foreign territory thanks to the provisions of Section 23.4 of Organic Law 6/1985 of July 1, of the Judiciary (as amended by Organic Law 1/2014 of 13 March).^2^ Thus, in Spain, health professionals have a legal obligation to inform the judicial authority of the possible existence of a criminal act. Therefore, professionals must assess risk of FGM and treat it as a child abuse-safeguarding and make referrals of under 18 years of age to the police. This is a legal requirement and responsibility.

Although legislative progress is an important step in eradicating this practice, it remains insufficient and strategies for social and cultural transformation must be designed and implemented beyond criminal and legal prohibitions. A guide for professionals published by the National Union of Family Associations (UNAF) in 2013, states that prevention should be a strategic priority line for the eradication of violence against girls and women; on the one hand, to “inform-sensitize” the population/families to facilitate a change in attitude towards FGM and, on the other hand, to “anticipate” cases of risk [7]. As proposed in EIGE’s report about ‘Female Genital Mutilation in the European Union and Croatia’, girls at risk of FGM are defined as minor girls (most commonly in the age range of 0–18) who come from FGM risk countries, or were born to parents (or one parent) who originate from countries where FGM is practised [18].

In 2015, the Spanish Ministry of Health, Social Services and Equality published a “Common protocol for health action against female genital mutilation” [12]. This protocol, based on the recommendations proposed previously by UNAF [7], develops, among others, the different actions necessary to be followed from the perspective of prevention and early detection of FGM.

It highlights that teams of health professionals, mainly at primary healthcare level, must have information about the network of community resources that facilitate the continuity of health and patient care. Also, in case of a possible situation of risk or when the FGM has occurred, they must know the existing tools for the health notification and, if necessary, the established channels for the communication of the fact to the rest of the sectors and agents involved (public entities of protection of minors, prosecution, judicial bodies, etc.) for an adequate follow-up of the case, putting in place the necessary protection measures or, if there is a crime, the report of such crime to the Justice. To this effect, the protocol insists on the need for training of professionals, which enables them to inform and guide women in case they need specific care and treatment either for themselves or for their daughters. Finally, the need for coordination between the different social care systems is also developed in order to be able to adopt effective actions in the prevention and awareness of FGM.

There is little research in our country about FGM and the knowledge, attitudes and practices (KAP) of professionals regarding this topic, but is even inferior in the primary healthcare context. As mentioned, primary healthcare professionals because of their proximity to the population have the opportunity and must identify the at-risk population, thus facilitating intervention in families and girls at risk. However, the revised literature indicates that there is a lack of knowledge related to the subject by health professionals, despite being a problem present in the Valencian community.

Kaplan-Marcusan et al. [19], in a study conducted between 2001 and 2004 in Catalonia, proved the existence of this social reality in primary healthcare centres, where at least 16% of the professionals had detected cases of women who had undergone FGM. However, the level of knowledge of the professionals on the subject was low, where less than half of the respondents knew the different types of FGM and only 22% knew the countries of origin of the practice.

In a systematic review by Zurynski et al. in 2015, only 18 studies were found from 2000 to 2014 in relation to the knowledge, attitudes and practices of health professionals. Although there are many publications on FGM, this systematic review evidences the lack of KAP research in Spain, and more specifically, in the Valencian Community [20]. This systematic review emphasized the need for accessible resources and evidence-based guidelines for professionals in order to provide culturally sensitive medical and psychological care to women and girls who have been mutilated.

The purpose of this study was to assess the perceptions, degree of knowledge and attitudes of primary healthcare professionals related to FGM.

## Methods

A crossectional descriptive study was conducted based on a self-administered questionnaire addressed to different groups of health professionals working in Primary Healthcare. Professionals from the health area Clínic-Malvarrosa of the city of Valencia, Spain, were included -general practitioners (GP), gynaecologists, paediatricians, nurses, midwives and social workers among others (one pharmacist and one psychologist)-. These groups were selected because of their “privileged” situation of closest action and with potentially greater involvement in the care of women and girls mutilated or at risk of FGM residing in the Valencian Community. The period of administration was from the first of March to the end of May 2017.

In the Valencian Healthcare System, primary healthcare professionals work in teams who attend the needs of the population assigned to a certain territory, known as “Healthcare Area”. This healthcare area is divided at the same time in different subareas with their own healthcare centre. In Clínic-Malvarrosa health area, primary healthcare is provided in thirty healthcare centres distributed throughout the health area by a multidisciplinary team (173 GPs, 168 nurses, 51 paediatricians, 19 midwives, 13 social workers and two gynaecologist, one pharmacist and a psychologist). The referral hospital for the whole Clínic-Malvarrosa area is called Hospital Clínico.

This health area serves a population of 340,481 people, of which 139,776 are women, 24,749 from 0 to 14 years old and 115,027 from 15 to 65 years of age according to the Patient Information System (data records from January 2017). According to the Valencian Community risk map of FGM, published by the Valencian Health Ministry in November 2016 [17], this health area belongs to one of the six areas with the highest number of girls and women at risk of FGM in the Valencian Community, accumulating 57% of the total of girls and women at risk of FGM. Specifically, this area is in the fifth position in the Valencian Community, with an estimation of 370 girls and women at risk of FGM (data based on the 2016 census).

The instrument used was a validated semi-structured questionnaire about “Knowledge, attitudes and practices of the health professionals relating FGM”.^3^ The questionnaire collected information on sociodemographic variables (age, gender, profession, speciality and workplace), degree of knowledge on FGM (identification and typology, aetiology, countries of prevalence, training received in the topic, knowledge of protocols and guidelines of action), cases of FGM and at-risk cases detected during consultations (previous experiences with women from countries where FGM is performed), interest on FGM (desire or not to act if a case detected) and attitudes versus FGM.

The attitudes explored were *educate-sensitise* -educate primary health professionals in FGM prevention and/or sensitize parents FGM consequences-; *report to authorities* -punitive and exemplary sentences to parents who perform FGM to their children and/or report to the authorities upon suspicion of FGM-; *control of children* -prevent girls to travel to their country of origin as to not to take any risk and/or perform routine check-ups of the female genitalia as a measure of control up to the age of eighteen-; or *ignore* any case of FGM or cases at risk of FGM.

A non-probabilistic sampling procedure was used since a nominal ratio of all professionals was not available for the random selection of the sample. Thus, the selection was non-probabilistic and by convenience sampling. However, this selection was made in a controlled basis, trying to guarantee the representability of the sample after estimating a proportion (percentage of health professionals in the primary healthcare area who correctly identify the types of FGM) taking as a reference the 40% obtained by Kaplan et al. in the study carried out in 2004. Considering this percentage as the expected proportion and applying an accuracy of 0.05 (5%) and a confidence level (1-α) of 90%, we required a sample of 260 cases, to which a 10% was added (taking into account those who potentially rejected to participate in the study). A total sample of 296 cases was finally estimated.

Additionally, we wanted to be exhaustive about specific groups such as paediatricians, social workers and midwives; although they were relatively small groups (in relation to the total set of medical professionals and nurses) they were considered as fundamental for the approach of this topic. Therefore, the questionnaire was handed over to eleven social workers working in different healthcare centres and two liaison social workers working in the hospital, representing the total social workers in the area. Twenty-five paediatricians and eleven midwives based in primary healthcare centres agreed to participate in the research, reaching more than a 50% of response in both groups.

The questionnaire was personally distributed during different educational and organizational meetings of the healthcare area professionals, and even contacting each centre independently with the purpose to reach the smaller groups as mentioned earlier. In order to obtain the highest percentage possible of responsiveness, the questionnaire was administered in paper form instead of self-administered e-mail survey (since it was not possible to have an official list of the same, and knowing the low rate of response of other studies). Different time slots and flexibility were facilitated for the distribution and verbal reminders (via telephone or in person) were used for collection.

Precise oral instructions were provided by the research team prior to completing the document highlighting the voluntary nature of participation. The purpose of the research was presented orally in an understandable manner to the participants, in the same way as the importance of their collaboration, the institution availing the study and the risks and potential benefits that could be obtained from it.

The whole research project had been approved by the Ethics Committee in Human Research of the Ethics Commission in Experimental Research of the University of Valencia, Spain. All the questionnaires were anonymous and participants could not be identified in any way. To that end, a single key number was assigned to each questionnaire.

### Statistical analysis

The answers provided by the participants were computed in a safe database for upkeep and debugging, making a first descriptive analysis of the variables under study and the subsequent assessment of association between them.

Categorical variables were described by the number of subjects and in absolute and relative frequencies including a 95% of confidence interval (CI). For the description of the continuous variables we used the mean, the standard deviations, 95% CI of the mean, the median, the 25th and 75th percentiles, the minimum and the maximum and total number of valid values.

In order to compare different groups of subjects, different statistical techniques were used according to the particulars of each variable and the number of groups to compare. If the variables, whether explanatory or response values, were categorical, the statistical analysis was made by using the Chi Square test with Fisher correction when necessary. Fisher’s exact test is used when more than 20% of the cells in the table have a frequency less than 5.

If the explanatory variable was categorical and the response variable was quantitative we applied the Student’s T test or ANOVA (for two or more groups respectively), after verification of the existence of a normal distribution, by means of the Kolmogorov-Smirnoff test. In the event that such a distribution is not corroborated, non-parametric Wilcoxon or Mann-Whitney tests were performed, as appropriate.

For the identification of the characteristics of the professionals which may influence knowledge about FGM, detection of cases and attitudes towards FGM, a logistical regression model was constructed, where the professional group (adult care –GPs and nurses-, maternal and child care –midwives, gynaecologists, paediatricians-, and social workers and others), age, gender and training received were used as independent variables and the knowledge of typology and countries of prevalence of FGM and the knowledge of the existence of protocols of action, detection of cases and attitudes (educate-sensitise, report to authorities, educate and report to the authorities, children regular controls) as dependent variables.

All analyses were conducted using STATA statistical package version 13.

## Results

Of a total of primary healthcare professionals of the healthcare area, a sample of 321 (75.1%) answered questionnaires was obtained; representing 71.1% (n123) of the total of professionals of family medicine (FM) in the department, 49% (n25) of all the paediatricians, 86.9% (n146) of nursing professionals, 57.9% (n11) of midwives and 100% (n13) of the group of social work professionals.

More than 70% of the respondents were women and the mean age of them was slightly lower (49.63) than that of men (53.13), with a minimum age of 22 and a maximum of 68 years old. The sociodemographic characteristics of the sample as well as professional knowledge, attitudes and practices in relationship to FGM are shown in Table 2 and are detailed by gender, age and professional group in Table 3.

**Table 2:** Sociodemographic characteristics of the sample and knowledge, attitudes and practices related to FGM

	N	%
Survey respondents	321	100
Profession		
GPs	123	38.3
Gynaecologist	1	0.3
Paediatricians	25	7.8
Nurses	146	45.5
Midwives	11	3.4
Social workers	13	4.1
Other	2	0.6
Gender		
Male	77	24
Female	230	71.7
Other	2	0.6
No answer	12	3.7
Age (years)		
≤ 35	43	13.4
36–50	69	21. 5
> 50	181	56.4
No answer	28	8.7
Training received	48	15
Proper training^a^	3	6.25
Correct identification		
Types of FGM	73	22.7
Countries of prevalence	16	**5**
Legislation	93	29
Reasons for conducting FGM		
Tradition and customs	120	37.4
Religious reasons	24	7.5
Tradition and religious reasons	130	40.5
Tradition and marriage opportunities	15	4.6
Other^b^	32	10
Detection of cases of FGM	15	4.7
Correctly identify cases at risk of FGM	109	34
Attitudes^c^		
Educate and sensitize	285	88.8
Condemn and report	131	40.8
Educate and report	113	35.2
Control	114	35.5

^a^Of those who responded having received any training, the ones who correctly identified types of FGM and countries of prevalence

^b^Other combinations, don’t know and don’t answer

^c^“Educate and sensitize”: educate primary health professionals in FGM prevention and/or sensitize parents FGM consequences. “Condemn and report”: punitive and exemplary sentences to parents who perform FGM to their children and/or report to the authorities upon suspicion of FGM. “Educate and report”: both previous options combined. “Control”: prevent girls to travel to their country of origin as to not to take any risk and/or perform routine check-ups of the female genitalia as a measure of control up to the age of eighteen

**Table 3:** Knowledge, attitudes and detection of cases according to gender, age and profession

	Age (*n* 293)							Gender (*n* 307)				Professionals (*n* 321)										
	≤35 (n 43)		36–50 (n 69)		> 50 (n 181)		*p*	Male (n 77)		Female (n 230)		GPs (n 123)		Paediatricians (n 25)		Nurses (n 146)		Midwives (n 11)		Social workers (n 13)		*p*
	N	%	N	%	N	%		N	%	N	%	N	%	N	%	N	%	N	%	N	%	
Knowledge																						
Correctly identify																						
Concept and types of FGM	9	20.9	16	23.2	43	23.8		17	22.1	54	23.5	29	23.6	8	32	25	17.1	8	72.7	3	23	0.001
Prevalence countries	1	2.3	4	5.8	8	4.4		5	6.5	10	4.4	13	37.2	6	17.1	13	37.1	1	2.7	2	5.7	
Legislation	8	18.6	16	23.2	59	32.6		21	27.3	66	28.7	40	43	10	10.7	33	35.5	6	6.5	3	3.2	
Protocol of action	3	7	6	8.70	12	6.63		4	5.2	18	7.8	3	2.4	7	28.0	3	2.1	5	45.5	3	23.1	< 0.001
Reasons for conducting FGM						< 0.001																
Tradition and customs	5	11.6	27	39.1	71	39.2		28	36.4	85	37	53	44.2	8	6.7	49	40.8	4	3.3	5	4.2	
Religious reasons	11	25.6	5	7.3	6	3.3		5	6.5	19	8.3	8	33.3	1	4.2	15	62.5	0	0	0	0	
Tradition and religious reasons	20	46.5	28	40.6	74	40.9		31	40.3	93	40.4	45	34.6	14	10.7	59	45.4	3	2.3	7	5.4	
Tradition and marriage opportunities	3	7	3	4.4	9	5		4	5.2	11	4.8	5	33.3	0	0	8	53.3	2	13.3	0	0	
Other	4	9.3	6	8.7	21	11.6		9	11.7	22	9.6	12	37.5	2	6.3	15	46.9	2	6.3	1	3.1	
Training received	7	16.3	8	11.6	30	16.5		10	13	37	16.1	14	11.3	5	20	19	13	7	63.6	2	15.3	0.006
Proper training^a^	0	0	0	0	3	10		0	0	3	8.1	1	0.8	0	0	0	0	2	18.2	0	0	< 0.001
Attitudes																						
Educate and sensitize	39	90.7	59	85.5	163	90.1		68	88.3	206	89.6	110	89.4	24	96	124	84.9	11	100	13	100	
Condemn and report	25	58.1	26	37.7	71	39.2		31	40.3	93	40.4	46	39.8	11	44	62	42.5	3	27.3	5	38.5	
Educate and report	21	48.8	21	30.4	64	35.4		27	35.1	81	35.2	45	36.6	10	40	49	33.6	3	27.3	5	38.5	
Control	10	23.3	30	43.5	66	36.5		28	36.4	82	35.7	46	37.4	13	52	46	31.5	2	18.2	4	30.8	
Detection																						
Have detected any case of FGM	2	4.7	3	4.4	8	4.4		3	3.9	11	4.8	4	3.3	1	4	5	3.4	4	36.4	1	7.7	< 0.05
Correctly identify cases at risk	17	39.5	21	30.4	63	34.8		30	39	78	33.9	35	28.5	11	44	51	34.9	6	54.6	5	38.5	

^a^Of those who responded having received any training, the ones who correctly identified types of FGM and countries of prevalence

Only 15% (n48) of the professionals answered that they had received training on FGM. On examining per disciplines, more than 60% (n7) of the midwives reported that they had received training followed by the 20% of paediatricians (n5) and 15.4% (n2) of social workers. On the other hand, GPs (n14) and nurses (n19) were the groups who reported to have received less training, with an 11.4% and a 13% respectively.

However, of those who responded that they had received training, only 6.3% (n3), two midwives and one GP, correctly identified the types of FGM and correctly pointed out the countries where this practice is carried out, considering these two items the most relevant in relation to the knowledge about the practice of FGM. Bivariate analysis found this fact statistically significant (*p* < 0.001). For the overall sample, the rate of correct identification of both items at time (typology and countries of prevalence) was even lower (2.2%, n7).

Focusing on the correct identification of the types of FGM and continuing with the total sample, 22.7% (n73) correctly identified the existence of the 3 types of FGM, although the great majority of the respondents (45.8%, n147) answered that FGM consists only in the total removal of the clitoris. Statistically significant differences were found between the different groups of professionals, the midwives being the group with the most knowledge about the types of FGM (*p* < 0.001).

As for countries where FGM is practiced, only 5% (n16) of the total sample correctly identified the geographical area. Almost a dozen (8.4%, n27) associated the practice of FGM exclusively to Muslim countries, 5.3% (n17) to the entire African continent, and others (49.5%, n159) associated it to only a few African countries. Less than one third (29%, n93) of the respondents correctly identified the current legislation in Spain; the same percentage as those who responded not knowing the current legislation (28%, n90). On the other hand, 38.3% (n123) think that FGM is a crime only if it is performed in Spain and the rest (4.4%, n14) responded that FGM is not legislated in Spain.

No differences were obtained by gender but there was a significant association between the reasons why FGM is performed and the age of the participants (*p* < 0.001). The belief that FGM is due to religious reasons is more frequent among the professionals in the younger group, while tradition and customs as a motive for the practice of FGM is higher among the group of professionals over 50 years old.

Of the surveyed professionals only 4.7% (n15) answered that they had ever found a case of FGM during their professional practice. Midwives, in proportion, had detected more cases of women and girls with FGM (36.4%) followed by social workers (7.7%). Of those who had never found any case, 19.3% (n58) responded not to attend to a population at risk of FGM in their professional practice, 30.9% (n93) responded to attend to a population at risk but had not found any cases and 7.3% (n22) admitted that in the framework of their activity it was not appropriate to ask about FGM. The rest (42.5%) did not justify why they had not found any case of FGM during their professional practice.

The detection of the cases was divided into detection in girls under 18 and over 18 years old. Of the total of fifteen cases found, eleven were over 18 years old; of which 72.7% (n8) were detected during a physical examination, two cases (18.2%) through a clinical interview and only one case through a third-party complaint. Screening was performed by pregnancy control (9.1%, n1), gynaecological examination (36.4%, n4) and by examination of another cause in the remaining five cases (45.5%). One person did not answer the question. The detection was casual, since in no case the exploration was due to suspicion of FGM. In relation to the action taken in case FGM was detected, one person decided to ignore it (9.1%) and the rest decided to act, basically coordinating with other professionals (45.5%, n5) or addressing the issue in the consultation and asking if they have any other daughters (preventing girls at risk of FGM in the same family unit) in the other 45.5% (n5) of the respondents. However, none of the respondents reported the findings.

With regard to the detection of FGM in girls under 18 years old, only 4 cases were detected through an interview and physical examination in the same proportion (50%), and no case by the complaint of a third party. When the detection was performed after physical examination, it was in both cases by pregnancy control in underaged women. They were casual findings not related to exploration because of suspicion of FGM. Of the two respondents who answered the question about the attitude towards the detection of a case in a minor, the two of them answered that they co-worked with other professionals. None of them addressed the issue in the consultation nor reported the situation found.

Furthermore, for the detection of cases at risk of FGM, 34% (n109) of respondents correctly answered the question of what a case of FGM risk is. Ten percent of the sample (n33) considered every African girl at risk and another 5.3% (n17) answered any African girl who travelled to her country of origin for holidays. The characteristics of the professionals who may have had an influence on the detection of cases, their attitudes and knowledge are shown in Table 4.

**Table 4:** Logistic regression model on knowledge, attitudes and detection of cases related to FGM and different independent variables

	Detection		Attitudes, Measures Proposed				Knowledge	
	Cases of FGM	Cases at risk of FGM	Educate and sensitize	Condemn and report	Educate and report	Control	Protocol of action	Correct identification of types and countries of prevalence of FGM
Gender								
Male	1	1	1	1	1	1	1	1
Female	0.8 (0.19–3.30)	0.9 (0.51–1.60)	1.1 (0.49–2.71)	0.9 (0.55–1.70)	0.9 (0.55–1.74)	1.02 (0.58–1.81)	1.1 (0.26–4.83)	0.7 (0.13–4.68)
Age (years)								
≤ 35	1	1	1	1	1	1	1	1
36–50	0.4 (0.06–3.91)	0.6 (0.29–1.55)	0.6 (0.17–2.16)	0.4 (0.19–0.98)*	0.4 (0.20–1.03)	2.8 (1.18–6.99)*	1.003 (0.18–5.49)	–
> 50	0.6 (0.11–3.43)	0.8 (0.41–1.69)	0.9 (0.30–3.07)	0.4 (0.21–0.87)*	0.5 (0.27–1.10)	2.1 (0.94–4.74)	0.4 (0.08–2.24)	–
Professional groups								
FM and nursing	1	1	1	1	1	1	1	1
Midwifery, paediatrics and gynaecology	5.1 (1.37–19.4)*	1.6 (0.80–3.42)	5.5 (0.70–43.57)	0.7 (0.37–1.66)	0.9 (0.45–2.02)	1.3 (0.62–2.71)	26.38 (7.57–91.93)*	7.4 (1.39–39.43)*
Social work and others	4.6 (0.47–46.22)	1.58 (0.47–5.26)	–	1.1 (0.33–3.68)	1.4 (0.44–4.88)	1.06 (0.32–4.40)	10.5 (1.59–70.54)*	–
Training								
Not received	1	1	1	1	1	1	1	
Received	8.6 (1.14–64.9)	0.7 (0.12–4.39)	0.3 (0.03–3.68)	3.5 (0.61–20.44)	1.9 (0.36–10.08)	0.7 (0.13–4.40)	195.6 (13.9–2750.1)*	
Professions								
GPs	1	1	1	1	1	1	1	1
Gynaecologists	–	–	–	–	–	–	–	–
Paediatricians	2.5 (0.21–30.11)	1.9 (0.77–4.97)	2.1 (0.25–18.09)	1.09 (0.44–2.70)	1.05 (0.39–2.62)	1.5 (0.62–3.82)	12.9 (3.0–55.67)	2.9 (0.24–34.82)
Nurses	1.8 (0.32–11.14)	1.6 (0.92–2.91)	0.4 (0.20–1.13)	0.8 (0.51–1.51)	0.6 (0.39–1.18)	0.8 (0.45–1.39)	0.7 (0.13–3.63)	0.5 (0.04–6.66)
Midwives	40.8 (5.17–322.1)*	3.3 (0.70–9.45)	–	0.4 (0.10–1.8)	0.5 (0.12–2.06)	0.3 (0.07–1.78)	26.8 (4.74–152.4)*	16.6 (1.46–188.60)*
Social workers	7.4 (0.54–102.4)	2.5 (0.70–9.45)	–	0.8 (0.22–3.06)	0.9 (0.25–3.52)	0.7 (1.19–2.71)	6.2 (0.84–45.95)	–

Results are expressed using Odds Ratio and CI 95% in parentheses

*p < 0.05 on comparison with the reference category

In the case of detection of girls or women at risk of FGM, 55.1% (n177) of the health professionals surveyed answered that they would always act, compared to 34% (n109) who responded not to act because they did not know how to. Less than a 3 % (n7) would only act in case of the girl travelling to her country of origin. Twelve people (3.7%) responded that they would not act since it was not within their competencies, and one person answered not to have had time to act. The rest (n15) did not answer the question.

As for the prioritization of the action to be performed after the detection of a risk case (excluding social workers), working in prevention obtained the highest number of responses as the first priority (40.5%). Secondly, examining the girl to check if she had FGM was mentioned (26.6%), followed by recording the findings in the clinical history (highest percentage as the third priority, 27.9%). Consulting/coordinating with other professionals was the 4th priority (27.9%) and putting the case to the knowledge of the Court was chosen mainly as the last option in the priority list of actions (40.5%).

The measures proposed by the professionals in order to prevent FGM in Spain were similar among health professionals of both sexes but differed significantly between the different ages. The measures of control and gynaecological screening were proposed mainly by the older groups (OR 2.8, 95% CI [1.18–6.99] in the 36–50 age group, and OR 2.1, 95% CI [0.94–4.74] in those over 50 years of age). The judicial measures and reporting to the police were proposed by the younger group. Training and sensitization obtained more uniform results among the different groups by age and gender (Additional file 1).

The 80% of the social workers surveyed agreed to prioritize contact with the paediatrics team as a first option, consulting/coordinating with other professionals obtained their maximum response rate as the second priority (40%) and explaining the consequences to the parents was prioritized as option 3 by 40% of the respondents. Reporting to the court was chosen with the lowest priority by 60% of the social workers.

Finally, in relation to the knowledge about the existence of some protocol of action, only 6.9% of the respondents admitted to know some protocol of action, obtaining statistically significant differences between the different professions and the knowledge of the existence of protocols of action. Midwives, paediatricians and social workers, with 45.5, 28 and 23.1% respectively, were the most aware of such protocols.

The detection of cases was similar between men and women, slightly superior in the male group, but mainly performed by professionals under 35 years old in the maternal and child care area (OR 5.1, 95% CI [1.37–19.4]) and social workers (OR 4.6, 95% CI [0.47–46.22]).

Considering the training variable as explanatory, and grouping the professionals into three categories; midwifery, paediatrics and gynaecology professionals detected statistically significant more cases of FGM (OR 5.1, 95% CI [1.37–19.4]) than social workers and others (OR 4.6, 95% CI [0.47–46.22]) and FM and nursing professionals. The midwifery, paediatrics and gynaecologists professional group ([OR 1.6, 95% CI (0.80–3.42]) and the social work professional group (OR 1.58, 95% CI [0.47–5.26]) did also detect more cases at risk than the family medicine and nursing professional group.

Subdividing the group of professionals, midwives had detected more cases of FGM (OR 40.8, 95% CI [5.17–322.1]) than social workers and paediatricians, this difference being statistically significant. In addition, midwives were the group that most correctly identified the types of FGM and the countries where this practice is performed (OR 16.6, 95% CI [1.46–188.60]), they better detect cases at risk (OR 3.3, 95% CI, [0.70–9.45]) and were aware of the existence of different protocols of action (OR 26.8, 95% CI [4.74–152.4]).

## Discussion

The results of our study show the existence of problems related to FGM in the population attended in consultations by the health professionals surveyed, since at least fifteen cases were detected. However, there was a lack of knowledge on the types of FGM, countries where it is carried out and the detection of cases of risk. This, added to the fact that more than one-third of the respondents admitted attending to the population at risk, suggests that there may be more underdiagnosed cases due to the ignorance of the existence of this practice but also because the strategies for its detection are not applied in many cases.

Thus, this detection figure of 4.7% is similar to the one detected by Kaplan et al. in 2001 in Catalonia (6%). Kaplan et al. detected, after carrying out different educational activities in the healthcare centres, an increase of FGM cases identified by GPs from 6 to 16% and paediatricians from 7 to 19% respectively between 2001 and 2004 [19]. The case detection figures are much higher in several European studies [21–26], although it is true that in these studies the sample is mainly of women’s specialists and are conducted in a hospital environment. Only in our study and the one carried out by Kaplan et al. in Catalonia the sample is entirely from the primary healthcare area. This may explain why European studies detection figures are higher than ours. Therefore, our study could only be compared to the one made by Kaplan et al., as it is the only study focusing on the primary healthcare area.

In 2013 in the UK, Purchase et al. surveyed 607 obstetricians and Relph surveyed 79 doctors and midwives [21, 22]. At least 87% of the obstetricians had been involved in the care of a mutilated girl or woman and more than 20% of them had known 10 cases. Almost 60 % of the professionals surveyed by Relph had found some case of FGM during their clinical practice. In Belgium 58% of the 333 gynaecologists surveyed by Leye had found cases of FGM, with infibulation being the most common type [23]. Cappon et al. in 2015, found that more than 15% of Flemish midwives were recently confronted with FGM but only 3.5% of them were aware of existing guidelines regarding FGM in their hospitals and only 20.2% was aware of the exact content of the law [24].

Similar are the figures in Sweden where 60% of women’s health professionals had found at least one case of FGM by detecting 39% of them long-term complications [25]. In Switzerland, 73% of French professionals had detected mutilated women in 2002 [26]. In Italy, the recorded figures are lower, however, the Caroppo et al. KAP study was targeted only to few professionals (physicians, social workers, psychologists and health assistants) working in asylum centres [27].

On the other hand, in our study, some professionals overestimated their knowledge about FGM. Only 6.3% was able to correctly identify typology of FGM and the countries of prevalence after answering to have been trained or to have carried out self-taught formation. The sources of training were mainly from NGOs and associations (16.7%) and services from official agencies (33.3%), although the largest percentage (35.4%) reported having received information through the media, television, newspapers and social networks.

Separating both items, less than a quarter of respondents correctly answered the existence of different types of FGM, and less than one-tenth of them correctly recognized the areas where FGM is practiced. More than a third of respondents believe that FGM is not legislated in Spain or does not know of its existence, 38% believes that it is only a crime if it is carried out in Spain and less than a tenth of the professionals of the centre were knowledgeable of the existence of official health protocols of action. We observe in this area an important gap of professional organisations communication towards the health professionals involved that needs urgent attention.

It is worth mentioning the recent elaboration and publication of the protocol of action of FGM by the Government of the Valencian Community, reinforcing the idea shown in this study on the importance of raising awareness about the existence and increase of population at risk in the Valencian Community. In spite of the institutional efforts made to train health professionals and the dissemination of the protocol and its performance (training courses for professionals, emails and corporate notifications about the recent protocol, guide of action in cases of girls traveling to their country of origin during holidays, etc.), this study shows the need to continue efforts for the prevention and eradication of this practice in our area.

Among the professionals who detected cases, the group of midwives was the only one who reported having detected more than one case of FGM during their professional practice. One of the midwives knew of at least ten cases of mutilated women, since she had the opportunity to meet other women who had undergone the same practice through one of her patients. Some professionals also commented on having worked in the United Kingdom (UK), where resources and guidance are published and updated frequently by the government to help to support the National Health Service (NHS) organisation developing new safeguarding policies and procedures for FGM.

These documents are developed in partnership with health and social care professionals and professional bodies and they can be used by health professionals from all sectors. Moreover, UK government encourages all organisations to ensure that their approach to safeguarding against FGM is multi-agency and multi-disciplinary [28]. They work with partners in social services and the police. A mandatory reporting duty for FGM requires regulated health and social care professionals and teachers in England and Wales to report known cases of FGM in under 18-year-olds to the police.

The maternal and infant stages are optimal stages for the detection of these girls or women, when in many occasions it will be their first contact with the health system. Addressing this issue in prenatal consultations requires great sensitivity, respect, empathy and confidentiality. The midwife, in this case, is usually the one that carries out a greater number of visits at this stage, creating a climate of trust and mutual respect more easily between professional and the woman, favouring the identification of women with FGM.

On the other hand, professionals should also practice prevention in the case of a new-born female, making sure that parents are aware of the criminal and health consequences that this entails for their daughter, should they wish to cut their daughter. The paediatrics team should also be contacted during gestation in case the pregnant woman with MGF is going to have a new-born female to continue raising awareness about preventing FGM in the girl. In any case, the detection of FGM must be registered in the available registry and computer systems, following the current algorithm published by the Health Department on the actions to be carried out in girls at risk.

Thus, midwives, gynaecologists, obstetricians, paediatricians and social workers need to acquire knowledge about different cultures and the sensitivity needed to ensure quality care and respect for women with FGM and their families. As early as 2013 in the United Kingdom, 84% of obstetricians surveyed were aware of the need to contact child protection services when a child was at risk of being mutilated [21].

Our study shows that more than a third of the professionals are able to detect cases of risk and that more than half of the respondents had an interest in acting if they located a child at risk. However, when the cases of FGM were detected in underaged girls, none of the professionals stated having reported the situation, and all the professional groups agreed that the last action they would take would be to bring the case to the attention of the Court. If detection of cases in women over 18 years are not reported and registered in the medical records, this may interfere with the provision of adequate care and prevention of FGM for the new-born daughter.

This fact suggests that professionals are not aware of the illegality of performing FGM in Spain as well as outside the territory for resident women, and that they have a duty and legal obligation to inform the judicial authority of the possible existence of a criminal act. This reinforces the idea of the need to distribute appropriate guidelines to the different healthcare, education workers and the police. The official ministry, professional’s organizations, and the specific health institutions by territory are responsible of developing these policies and guidelines and should provide training awareness-raising for all the professionals dealing with FGM. The importance of individual risk assessments cannot be underestimated. Assessing the risk that a girl might be exposed to because one or both her parents originate from countries where FGM is practised is one of the criteria to be used to assess risk and initiate, if considered, specific prevention and protection actions.

Nevertheless, the priority of the strategic line with a view to eradicate violence against women in general and FGM as a specific form of violence should be prevention and awareness of the population from a multicultural and integral approach, being the Primary Health Care a fundamental pillar in the health system, if the professionals are trained and sensitized with this health problem. To achieve this, a proposal could be to introduce FGM in the curricula of various disciplines at university and high school level, as WHO advised in 2001 [29]. In addition, it is essential to establish a coordination system with other administrations (educational, social, judicial and police) to complete the comprehensive and multidisciplinary approach to FGM, including any legal consequences that may arise.

In order to know the knowledge base and to describe the capacities and attitudes of the other two main pillars for the prevention and eradication of this practice, education professionals and security authorities have also been surveyed; the results will soon be published elsewhere. This line of research is broad and little explored and combined with the exploration of the needs and experiences of immigrant families in our region, it will lead to a better understanding of the necessary improvements of the different public sectors for the early detection, prevention, follow-up and intervention in women and girls with FGM.

With regard to the limitations of this study we believe that the lack of statistical significance in some of the trends observed on multivariate analysis was due to insufficient sample size in some of the variables studied as some groups of the healthcare area studied, such as midwives, gynaecologists and social workers do not contain a wide range of professionals. It should also be noted that initially, we did not receive a list of professionals. This would have facilitated the distribution of the questionnaires through random sampling and the calculation of the sample for its representativeness. It should also be indicated that the possible ambiguity in some questions of the questionnaire could lead to different interpretations and to different response criteria for the respondents, such as the response to “working in prevention of FGM”.

## Conclusions

This study demonstrates that the problem of FGM is present in the population attending primary healthcare services in our region. However, the professionals showed a profound lack of knowledge around concept, typology and countries of prevalence of FGM suggesting that, added to the fact that more than one-third of the respondents admitted attending to population at risk, there may be more underdiagnosed cases of FGM. The professionals also over-evaluated their degree of knowledge and less than a quarter of respondents correctly answered the existence of different types of FGM and less than one-tenth of them correctly recognized the areas where FGM is practiced. The detection of cases was mainly performed by professionals under 35 years old, those working in the field of midwifery, paediatrics and gynaecology followed by social workers. Less than 7 % admitted to know some protocol of action, but midwives, paediatricians and social workers were most aware of such protocols. Midwives were also the group that were mostly able to identify the types of FGM, the countries where this practice is performed and to detect cases at risk.

To guarantee a multidisciplinary, transcultural and respectful approach to FGM in our community, it is necessary to make the problem of FGM known to the population in general and to healthcare professionals and other public services professionals in particular, increasing their knowledge about this fact and focusing on the importance of prevention and early detection of cases at risk of FGM. It is healthcare professional’s duty to recognize this situation and to follow the right protocols of action in order to refer these women and their families to the most appropriate services and professionals that fit their needs, thus ensuring a multidisciplinary, positive and transcultural care for these families. Preventive and awareness-raising measures should also be undertaken to attempt to eradicate this practice in our area and worldwide.

The study is carried out in a particular area of the city of Valencia and although it can be thought that the situation may be extrapolated to the rest of the Community, due to its population heterogeneity, it would have to be confirmed in future research. Continuing to collect useful data is crucial for guiding public policies and programs as an essential part of the efforts to eradicate FGM.

## Additional file

Additional file 1: Healthcare professionals multivariate analysis. (PDF 662 kb)

## Footnotes

1 Ley Orgánica 10/1995, de 23 de noviembre, del Código Penal.

2 Ley Orgánica 11/2003, de 29 de septiembre, de medidas concretas en materia de seguridad ciudadana, violencia doméstica e integración social de los extranjeros.

3 Designed and validated questionnaire by Kaplan-Marcusan (Wassu-UAB Foundation), authorised for use by the author.
