# Supplementary material for: Knowledge, attitudes and practices of primary healthcare professionals to female genital mutilation in Valencia, Spain: are we ready for this challenge?
Source: BMC Health Serv Res. 2018 Jul 24;18:579. doi: 10.1186/s12913-018-3396-z (PMC6057065; doi:10.1186/s12913-018-3396-z)
Supplement: Supplementary file 1 — Healthcare professionals multivariate analysis. (PDF 662 kb) [file 12913_2018_3396_MOESM1_ESM.pdf]

MULTIVARIATE ANALYSIS HEALTHCARE PROFESSIONAL.S

TRAINING VARIABLE

```
. tab Formacion


  Formacion |      Freq.     Percent        Cum.
------------+-----------------------------------
         No |        314       97.82       97.82
         Si |          7        2.18      100.00
------------+-----------------------------------
      Total |        321      100.00


. xi:logistic Formacion i.sexo i.edadgroup  i.I4Profesin
i.sexo            _Isexo_1-2          (naturally coded; _Isexo_1 omitted)
i.edadgroup       _Iedadgroup_1-3     (naturally coded; _Iedadgroup_1 omitted)
i.I4Profesin      _II4Profesi_1-11    (naturally coded; _II4Profesi_1 omitted)
note: _Iedadgroup_2 != 0 predicts failure perfectly
      _Iedadgroup_2 dropped and 67 obs not used


note: _Iedadgroup_3 != 1 predicts failure perfectly
      _Iedadgroup_3 dropped and 41 obs not used


note: _II4Profesi_2 != 0 predicts failure perfectly
      _II4Profesi_2 dropped and 1 obs not used


note: _II4Profesi_6 != 0 predicts failure perfectly
      _II4Profesi_6 dropped and 5 obs not used


note: _II4Profesi_11 != 0 predicts failure perfectly
      _II4Profesi_11 dropped and 1 obs not used
```

```
Logistic regression                               Number of obs     =        171
                                                  LR chi2(4)        =       7.17
                                                  Prob > chi2       =     0.1273
Log likelihood = -22.409645                       Pseudo R2         =     0.1379

--------------------------------------------------------------------------------
     Formacion | Odds Ratio   Std. Err.      z    P>|z|     [95% Conf. Interval]
---------------+----------------------------------------------------------------
      _Isexo_2 |   .5187199   .5390697    -0.63   0.528      .067661    3.976745
 _Iedadgroup_2 |          1  (omitted)
 _Iedadgroup_3 |          1  (omitted)
 _II4Profesi_2 |          1  (omitted)
 _II4Profesi_3 |    2.90293   3.680194     0.84   0.401     .2419494    34.82961
 _II4Profesi_4 |   .5630458   .7098329    -0.46   0.649     .0475809    6.662776
 _II4Profesi_5 |   16.61525   20.59416     2.27   0.023     1.463745    188.6029
 _II4Profesi_6 |          1  (omitted)
_II4Profesi_11 |          1  (omitted)
         _cons |   .0386758   .0326466    -3.85   0.000     .0073949    .2022773
--------------------------------------------------------------------------------
```

CASES DETECTED

```
. tab Deteccion_casos

Deteccion_c |
       asos |      Freq.     Percent        Cum.
------------+-----------------------------------
         No |        306       95.33       95.33
         Si |         15        4.67      100.00
------------+-----------------------------------
      Total |        321      100.00

. xi:logistic Deteccion_casos i.sexo i.edadgroup  i.I4Profesin
i.sexo            _Isexo_1-2          (naturally coded; _Isexo_1 omitted)
i.edadgroup       _Iedadgroup_1-3     (naturally coded; _Iedadgroup_1 omitted)
i.I4Profesin      _II4Profesi_1-11    (naturally coded; _II4Profesi_1 omitted)
note: _II4Profesi_2 != 0 predicts failure perfectly
```

```
      _II4Profesi_2 dropped and 1 obs not used

note: _II4Profesi_11 != 0 predicts failure perfectly
      _II4Profesi_11 dropped and 1 obs not used


Logistic regression                               Number of obs   =        284
                                                  LR chi2(7)      =      15.37
                                                  Prob > chi2     =     0.0316
Log likelihood = -42.028448                       Pseudo R2       =     0.1546

--------------------------------------------------------------------------------
Deteccion_casos | Odds Ratio   Std. Err.      z    P>|z|     [95% Conf. Interval]
----------------+---------------------------------------------------------------
       _Isexo_2 |   .4641893   .3652123    -0.98   0.329     .0993097    2.169695
  _Iedadgroup_2 |   .6586704   .7296886    -0.38   0.706     .0751086    5.776259
  _Iedadgroup_3 |   .8619662   .7626295    -0.17   0.867     .1521896    4.881976
  _II4Profesi_2 |          1  (omitted)
  _II4Profesi_3 |   2.558102   3.218139     0.75   0.455     .2173156    30.11237
  _II4Profesi_4 |    1.89473   1.713295     0.71   0.480     .3219992     11.1491
  _II4Profesi_5 |   40.84208   43.03854     3.52   0.000     5.177608     322.171
  _II4Profesi_6 |   7.450518   9.962604     1.50   0.133     .5419834    102.4205
 _II4Profesi_11 |          1  (omitted)
          _cons |   .0348258   .0389862    -3.00   0.003     .0038816    .3124557
--------------------------------------------------------------------------------
```

ATTITUDES

Educate and Sensitize

. tab I121

```
   Formar y |
sensibiliza |
          r |      Freq.     Percent        Cum.
------------+-----------------------------------
          0 |         36       11.21       11.21
```

```
          1 |        285       88.79      100.00
------------+-----------------------------------
      Total |        321      100.00

. xi:logistic I121 i.sexo i.edadgroup  i.I4Profesin
i.sexo            _Isexo_1-2          (naturally coded; _Isexo_1 omitted)
i.edadgroup       _Iedadgroup_1-3     (naturally coded; _Iedadgroup_1 omitted)
i.I4Profesin      _II4Profesi_1-11    (naturally coded; _II4Profesi_1 omitted)
note: _II4Profesi_2 != 0 predicts success perfectly
      _II4Profesi_2 dropped and 1 obs not used

note: _II4Profesi_5 != 0 predicts success perfectly
      _II4Profesi_5 dropped and 11 obs not used

note: _II4Profesi_6 != 0 predicts success perfectly
      _II4Profesi_6 dropped and 11 obs not used

note: _II4Profesi_11 != 0 predicts success perfectly
      _II4Profesi_11 dropped and 1 obs not used


Logistic regression                               Number of obs   =        262
                                                  LR chi2(5)      =       5.85
                                                  Prob > chi2     =     0.3211
Log likelihood = -92.329419                       Pseudo R2       =     0.0307

------------------------------------------------------------------------------
        I121 | Odds Ratio   Std. Err.      z    P>|z|     [95% Conf. Interval]
-------------+----------------------------------------------------------------
    _Isexo_2 |   1.388033   .6154235     0.74   0.460     .5820977    3.309817
 _Iedadgroup_2 |   .6019141   .3887362    -0.79   0.432     .1697463    2.134366
 _Iedadgroup_3 |   .8404449   .4992279    -0.29   0.770     .2623565     2.69232
 _II4Profesi_2 |          1  (omitted)
 _II4Profesi_3 |    2.16371   2.344528     0.71   0.476     .2587401    18.09398
 _II4Profesi_4 |   .4803501   .2102777    -1.67   0.094     .2036745    1.132868
 _II4Profesi_5 |          1  (omitted)
 _II4Profesi_6 |          1  (omitted)
```

```
_II4Profesi_11 |          1  (omitted)
         _cons |   10.90358   7.513997     3.47   0.001     2.824747    42.08803
---------------+----------------------------------------------------------------
```

Condemn and report

```
. tab I122

 Condenar y |
  denunciar |      Freq.     Percent        Cum.
------------+-----------------------------------
          0 |        190       59.19       59.19
          1 |        131       40.81      100.00
------------+-----------------------------------
      Total |        321      100.00

. xi:logistic I122 i.sexo i.edadgroup  i.I4Profesin
i.sexo            _Isexo_1-2          (naturally coded; _Isexo_1 omitted)
i.edadgroup       _Iedadgroup_1-3     (naturally coded; _Iedadgroup_1 omitted)
i.I4Profesin      _II4Profesi_1-11    (naturally coded; _II4Profesi_1 omitted)
note: _II4Profesi_2 != 0 predicts failure perfectly
      _II4Profesi_2 dropped and 1 obs not used

note: _II4Profesi_11 != 0 predicts success perfectly
      _II4Profesi_11 dropped and 1 obs not used


Logistic regression                               Number of obs   =        284
                                                  LR chi2(7)      =       6.98
                                                  Prob > chi2     =     0.4305
Log likelihood = -189.61955                       Pseudo R2       =     0.0181

--------------------------------------------------------------------------------
          I122 | Odds Ratio   Std. Err.      z    P>|z|     [95% Conf. Interval]
---------------+----------------------------------------------------------------
      _Isexo_2 |   .9984929   .2914242    -0.01   0.996     .5635207    1.769213
 _Iedadgroup_2 |   .4365718   .1793359    -2.02   0.044     .1951629    .9765939
```

```
 _Iedadgroup_3 |   .4447597   .1596391    -2.26   0.024     .2200903    .8987732
 _II4Profesi_2 |          1  (omitted)
 _II4Profesi_3 |   1.096392   .5060669     0.20   0.842     .4436828    2.709312
 _II4Profesi_4 |   .8802505   .2426748    -0.46   0.644     .5127904    1.511028
 _II4Profesi_5 |    .452972   .3278236    -1.09   0.274     .1096584    1.871115
 _II4Profesi_6 |   .8228683   .5518824    -0.29   0.771      .221027    3.063482
_II4Profesi_11 |          1  (omitted)
         _cons |   1.579422   .6656278     1.08   0.278     .6914642    3.607668
------------------------------------------------------------------------------
```

Educate and condemn

```
. tab I123b

      I123b |      Freq.     Percent        Cum.
------------+-----------------------------------
          0 |        208       64.80       64.80
          1 |        113       35.20      100.00
------------+-----------------------------------
      Total |        321      100.00

. xi:logistic I123b i.sexo i.edadgroup  i.I4Profesin
i.sexo            _Isexo_1-2          (naturally coded; _Isexo_1 omitted)
i.edadgroup       _Iedadgroup_1-3     (naturally coded; _Iedadgroup_1 omitted)
i.I4Profesin      _II4Profesi_1-11    (naturally coded; _II4Profesi_1 omitted)
note: _II4Profesi_2 != 0 predicts failure perfectly
      _II4Profesi_2 dropped and 1 obs not used

note: _II4Profesi_11 != 0 predicts success perfectly
      _II4Profesi_11 dropped and 1 obs not used


Logistic regression                               Number of obs   =        284
                                                  LR chi2(7)      =       6.28
                                                  Prob > chi2     =     0.5069
Log likelihood = -182.86129                       Pseudo R2       =     0.0169

------------------------------------------------------------------------------
         I123b | Odds Ratio   Std. Err.      z    P>|z|     [95% Conf. Interval]
```

```
----------------+----------------------------------------------------------------
       _Isexo_2 |   1.064534   .3190819     0.21   0.835     .5915915    1.915568
   _Iedadgroup_2 |   .4482597   .1869535    -1.92   0.054     .1979353    1.015164
   _Iedadgroup_3 |   .5321901   .1903714    -1.76   0.078     .2639859    1.072884
   _II4Profesi_2 |          1  (omitted)
   _II4Profesi_3 |   1.052502   .4903058     0.11   0.913     .4223694    2.622728
   _II4Profesi_4 |   .6794556   .1923856    -1.36   0.172     .3900732    1.183521
   _II4Profesi_5 |    .501523   .3619097    -0.96   0.339     .1219118    2.063175
   _II4Profesi_6 |   .9440261   .6347321    -0.09   0.932     .2527342    3.526175
  _II4Profesi_11 |          1  (omitted)
           _cons |   1.178943   .4977756     0.39   0.697     .5153434    2.697049
---------------------------------------------------------------------------------
```

Control

```
. tab I124

    Control |      Freq.     Percent        Cum.
------------+-----------------------------------
          0 |        207       64.49       64.49
          1 |        114       35.51      100.00
------------+-----------------------------------
      Total |        321      100.00

. xi:logistic I124 i.sexo i.edadgroup  i.I4Profesin
i.sexo            _Isexo_1-2          (naturally coded; _Isexo_1 omitted)
i.edadgroup       _Iedadgroup_1-3     (naturally coded; _Iedadgroup_1 omitted)
i.I4Profesin      _II4Profesi_1-11    (naturally coded; _II4Profesi_1 omitted)
note: _II4Profesi_2 != 0 predicts success perfectly
      _II4Profesi_2 dropped and 1 obs not used

note: _II4Profesi_11 != 0 predicts success perfectly
      _II4Profesi_11 dropped and 1 obs not used


Logistic regression                             Number of obs     =        284
                                                LR chi2(7)        =      10.03
```

```
                                                  Prob > chi2     =     0.1869
Log likelihood = -180.98922                       Pseudo R2       =     0.0270

--------------------------------------------------------------------------------
          I124 | Odds Ratio   Std. Err.      z    P>|z|     [95% Conf. Interval]
---------------+----------------------------------------------------------------
      _Isexo_2 |   1.172498   .3536163     0.53   0.598     .6492268    2.117522
 _Iedadgroup_2 |   2.809282   1.283579     2.26   0.024     1.147298    6.878832
 _Iedadgroup_3 |   2.016541   .8370929     1.69   0.091     .8938444    4.549379
 _II4Profesi_2 |          1  (omitted)
 _II4Profesi_3 |   1.544104   .7142649     0.94   0.348     .6236362    3.823153
 _II4Profesi_4 |   .8008109   .2267999    -0.78   0.433     .4596834    1.395087
 _II4Profesi_5 |   .3575289   .2932245    -1.25   0.210     .0716481    1.784093
 _II4Profesi_6 |   .7224628   .4876513    -0.48   0.630     .1924277    2.712461
_II4Profesi_11 |          1  (omitted)
         _cons |   .2818755   .1337123    -2.67   0.008     .1112438    .7142315
--------------------------------------------------------------------------------
```

CORRECTLY IDENTIFY CASES AT RISK

```
. tab DetectaCRcorrecto

DetectaCRco |
     rrecto |      Freq.     Percent        Cum.
------------+-----------------------------------
         No |        212       66.04       66.04
         Si |        109       33.96      100.00
------------+-----------------------------------
      Total |        321      100.00

. xi:logistic DetectaCRcorrecto i.sexo i.edadgroup  i.I4Profesin
i.sexo            _Isexo_1-2          (naturally coded; _Isexo_1 omitted)
i.edadgroup       _Iedadgroup_1-3     (naturally coded; _Iedadgroup_1 omitted)
i.I4Profesin      _II4Profesi_1-11    (naturally coded; _II4Profesi_1 omitted)
note: _II4Profesi_2 != 0 predicts failure perfectly
      _II4Profesi_2 dropped and 1 obs not used

note: _II4Profesi_11 != 0 predicts failure perfectly
```

```
      _II4Profesi_11 dropped and 1 obs not used



Logistic regression                               Number of obs   =        284
                                                  LR chi2(7)      =       7.08
                                                  Prob > chi2     =     0.4211
Log likelihood = -180.70578                       Pseudo R2       =     0.0192

------------------------------------------------------------------------------
DetectaCRcorrecto | Odds Ratio   Std. Err.      z    P>|z|     [95% Conf. Interval]
------------------+-----------------------------------------------------------
         _Isexo_2 |   .7786095   .2353807    -0.83   0.408     .4305205    1.408139
    _Iedadgroup_2 |   .6976788   .2961277    -0.85   0.396     .3036446    1.603044
    _Iedadgroup_3 |   .8928601   .3258448    -0.31   0.756     .4366583    1.825682
    _II4Profesi_2 |          1  (omitted)
    _II4Profesi_3 |   1.965921   .9305625     1.43   0.153     .7774141     4.97141
    _II4Profesi_4 |   1.639373   .4804858     1.69   0.092     .9229921    2.911773
    _II4Profesi_5 |   3.333966   2.191137     1.83   0.067     .9194701    12.08884
    _II4Profesi_6 |   2.581355   1.709585     1.43   0.152     .7048754    9.453296
   _II4Profesi_11 |          1  (omitted)
            _cons |   .5188295   .2248849    -1.51   0.130     .2218575     1.21332
------------------------------------------------------------------------------
```

KNOWLEDGE OF PROTOCOL OF ACTION

```
. tab protocolo

  protocolo |      Freq.     Percent        Cum.
------------+-----------------------------------
         No |        299       93.15       93.15
         Si |         22        6.85      100.00
------------+-----------------------------------
      Total |        321      100.00
```

```
. xi:logistic protocolo i.sexo i.edadgroup  i.I4Profesin

i.sexo            _Isexo_1-2          (naturally coded; _Isexo_1 omitted)

i.edadgroup       _Iedadgroup_1-3     (naturally coded; _Iedadgroup_1 omitted)

i.I4Profesin      _II4Profesi_1-11    (naturally coded; _II4Profesi_1 omitted)

note: _II4Profesi_2 != 0 predicts success perfectly

      _II4Profesi_2 dropped and 1 obs not used


note: _II4Profesi_11 != 0 predicts failure perfectly

      _II4Profesi_11 dropped and 1 obs not used



Logistic regression                               Number of obs   =        284

                                                  LR chi2(7)      =      34.91

                                                  Prob > chi2     =     0.0000

Log likelihood = -54.889302                       Pseudo R2       =     0.2413


------------------------------------------------------------------------------

     protocolo | Odds Ratio   Std. Err.      z    P>|z|     [95% Conf. Interval]

---------------+--------------------------------------------------------------

      _Isexo_2 |   1.231757   .8946765     0.29   0.774     .2966619    5.114324

  _Iedadgroup_2 |   1.142987   .9822415     0.16   0.876     .2121045    6.159322

  _Iedadgroup_3 |   .7461473   .5811564    -0.38   0.707     .1621227    3.434039

  _II4Profesi_2 |          1  (omitted)

  _II4Profesi_3 |    12.9298   9.631518     3.44   0.001     3.002787    55.67485

  _II4Profesi_4 |   .7001704   .5885958    -0.42   0.672     .1347858    3.637168

  _II4Profesi_5 |   26.89961   23.80586     3.72   0.000     4.747247    152.4228

  _II4Profesi_6 |   6.241757   6.357464     1.80   0.072     .8478639    45.95022

 _II4Profesi_11 |          1  (omitted)

          _cons |    .030722   .0319279    -3.35   0.001     .0040072    .2355386
```

Multivariate ANALYSIS_ TRAINING VARIABLE AS INDEPENDENT

_Igruposani_1=Etapa adulto

_Igruposani_2=Etapa perimaternal

_Igruposani_3=Trabajador social y otros

TRAINING

```
. xi:logistic Formacion i.sexo i.edadgroup  i.gruposanitario
i.sexo            _Isexo_1-2          (naturally coded; _Isexo_1 omitted)
i.edadgroup       _Iedadgroup_1-3     (naturally coded; _Iedadgroup_1 omitted)
i.gruposanita~o   _Igruposani_1-3     (naturally coded; _Igruposani_1 omitted)
note: _Iedadgroup_2 != 0 predicts failure perfectly
      _Iedadgroup_2 dropped and 67 obs not used

note: _Iedadgroup_3 != 1 predicts failure perfectly
      _Iedadgroup_3 dropped and 41 obs not used

note: _Igruposani_3 != 0 predicts failure perfectly
      _Igruposani_3 dropped and 6 obs not used


Logistic regression                               Number of obs   =        172
                                                  LR chi2(2)      =       4.94
                                                  Prob > chi2     =     0.0847
Log likelihood = -23.560008                       Pseudo R2       =     0.0948

------------------------------------------------------------------------------
    Formacion | Odds Ratio   Std. Err.      z    P>|z|     [95% Conf. Interval]
-------------+----------------------------------------------------------------
     _Isexo_2 |   .7949539   .7191152    -0.25   0.800     .1350035    4.681001
_Iedadgroup_2 |          1  (omitted)
_Iedadgroup_3 |          1  (omitted)
_Igruposani_2 |    7.40517   6.318887     2.35   0.019     1.390587    39.43408
_Igruposani_3 |          1  (omitted)
        _cons |   .0239091    .019578    -4.56   0.000     .0048035    .1190066
------------------------------------------------------------------------------
```

CASES DETECTED

```
. xi:logistic Deteccion_casos i.sexo i.edadgroup  i.gruposanitario
i.sexo            _Isexo_1-2          (naturally coded; _Isexo_1 omitted)
i.edadgroup       _Iedadgroup_1-3     (naturally coded; _Iedadgroup_1 omitted)
i.gruposanita~o   _Igruposani_1-3     (naturally coded; _Igruposani_1 omitted)
```

```
Logistic regression                               Number of obs   =        286
                                                  LR chi2(5)      =       8.26
                                                  Prob > chi2     =     0.1426
Log likelihood =  -45.66879                       Pseudo R2       =     0.0829

------------------------------------------------------------------------------
Deteccion_casos | Odds Ratio   Std. Err.      z    P>|z|     [95% Conf. Interval]
----------------+-------------------------------------------------------------
       _Isexo_2 |   .8665918   .6184452    -0.20   0.841     .2139699    3.509752
  _Iedadgroup_2 |   .5003096    .530944    -0.65   0.514     .0625054     4.00461
  _Iedadgroup_3 |   .8166313   .6892449    -0.24   0.810     .1561721    4.270204
  _Igruposani_2 |   6.360202   4.070662     2.89   0.004     1.814195    22.29758
  _Igruposani_3 |   4.256411   4.930474     1.25   0.211     .4395842    41.21402
          _cons |   .0370475   .0363492    -3.36   0.001      .005415    .2534641
------------------------------------------------------------------------------

. xi:logistic Deteccion_casos i.sexo i.edadgroup  i.gruposanitario i.Formacion
i.sexo            _Isexo_1-2          (naturally coded; _Isexo_1 omitted)
i.edadgroup       _Iedadgroup_1-3     (naturally coded; _Iedadgroup_1 omitted)
i.gruposanita~o   _Igruposani_1-3     (naturally coded; _Igruposani_1 omitted)
i.Formacion       _IFormacion_0-1     (naturally coded; _IFormacion_0 omitted)

Logistic regression                               Number of obs   =        286
                                                  LR chi2(6)      =      11.89
                                                  Prob > chi2     =     0.0644
Log likelihood = -43.851512                       Pseudo R2       =     0.1194

------------------------------------------------------------------------------
Deteccion_casos | Odds Ratio   Std. Err.      z    P>|z|     [95% Conf. Interval]
----------------+-------------------------------------------------------------
       _Isexo_2 |   .7943933   .5773132    -0.32   0.751      .191178    3.300907
  _Iedadgroup_2 |   .4880637   .5184977    -0.68   0.500     .0608411     3.91522
  _Iedadgroup_3 |   .6265638   .5436059    -0.54   0.590       .11441    3.431362
  _Igruposani_2 |   5.161862   3.487362     2.43   0.015     1.373198    19.40347
  _Igruposani_3 |   4.662287   5.456939     1.32   0.188     .4702365    46.22549
  _IFormacion_1 |   8.629604   8.889133     2.09   0.036     1.146014    64.98179
```

```
         _cons |   .0430947   .0415951    -3.26   0.001     .0064989    .2857629
------------------------------------------------------------------------------
```

1.1 I12ACTIVIDADES

Formar y sensibilizar

```
. xi:logistic I121 i.sexo i.edadgroup  i.gruposanitario
i.sexo            _Isexo_1-2          (naturally coded; _Isexo_1 omitted)
i.edadgroup       _Iedadgroup_1-3     (naturally coded; _Iedadgroup_1 omitted)
i.gruposanita~o   _Igruposani_1-3     (naturally coded; _Igruposani_1 omitted)
note: _Igruposani_3 != 0 predicts success perfectly
      _Igruposani_3 dropped and 12 obs not used


Logistic regression                               Number of obs   =        274
                                                  LR chi2(4)      =       5.08
                                                  Prob > chi2     =     0.2794
Log likelihood = -94.190476                       Pseudo R2       =     0.0262

------------------------------------------------------------------------------
         I121 | Odds Ratio   Std. Err.      z    P>|z|     [95% Conf. Interval]
--------------+----------------------------------------------------------------
      _Isexo_2 |   1.190818   .5120576     0.41   0.685     .5126544    2.766089
_Iedadgroup_2 |   .6152497   .3949307    -0.76   0.449     .1748487    2.164912
_Iedadgroup_3 |   .9345678   .5494812    -0.12   0.908     .2952194    2.958535
_Igruposani_2 |   5.005007   5.175253     1.56   0.119     .6595523    37.98045
_Igruposani_3 |          1  (omitted)
        _cons |   7.193141   4.523492     3.14   0.002     2.097154    24.67214
------------------------------------------------------------------------------

. xi:logistic I121 i.sexo i.edadgroup  i.gruposanitario i.Formacion
i.sexo            _Isexo_1-2          (naturally coded; _Isexo_1 omitted)
i.edadgroup       _Iedadgroup_1-3     (naturally coded; _Iedadgroup_1 omitted)
i.gruposanita~o   _Igruposani_1-3     (naturally coded; _Igruposani_1 omitted)
i.Formacion       _IFormacion_0-1     (naturally coded; _IFormacion_0 omitted)
note: _Igruposani_3 != 0 predicts success perfectly
```

```
      _Igruposani_3 dropped and 12 obs not used


Logistic regression                               Number of obs   =        274
                                                  LR chi2(5)      =       5.71
                                                  Prob > chi2     =     0.3352
Log likelihood = -93.873234                       Pseudo R2       =     0.0295

------------------------------------------------------------------------------
        I121 | Odds Ratio   Std. Err.      z    P>|z|     [95% Conf. Interval]
-------------+----------------------------------------------------------------
     _Isexo_2 |   1.161916   .5031317     0.35   0.729     .4972653    2.714946
_Iedadgroup_2 |   .6149053   .3948017    -0.76   0.449     .1746996    2.164335
_Iedadgroup_3 |   .9660572   .5706342    -0.06   0.953     .3035372    3.074636
_Igruposani_2 |   5.552191   5.836293     1.63   0.103     .7074691    43.57339
_Igruposani_3 |          1  (omitted)
_IFormacion_1 |    .355171   .4241773    -0.87   0.386     .0341866     3.68994
        _cons |    7.31178   4.608684     3.16   0.002     2.125704    25.15032
------------------------------------------------------------------------------
```

Condenar y denunciar

```
. xi:logistic I122 i.sexo i.edadgroup  i.gruposanitario
i.sexo            _Isexo_1-2          (naturally coded; _Isexo_1 omitted)
i.edadgroup       _Iedadgroup_1-3     (naturally coded; _Iedadgroup_1 omitted)
i.gruposanita~o   _Igruposani_1-3     (naturally coded; _Igruposani_1 omitted)

Logistic regression                               Number of obs   =        286
                                                  LR chi2(5)      =       5.52
                                                  Prob > chi2     =     0.3554
Log likelihood = -191.76292                       Pseudo R2       =     0.0142

------------------------------------------------------------------------------
        I122 | Odds Ratio   Std. Err.      z    P>|z|     [95% Conf. Interval]
-------------+----------------------------------------------------------------
     _Isexo_2 |   .9708693   .2749439    -0.10   0.917     .5573217     1.69128
_Iedadgroup_2 |   .4447866   .1815034    -1.99   0.047     .1998956    .9896923
_Iedadgroup_3 |   .4559695   .1619468    -2.21   0.027     .2273078     .914655
_Igruposani_2 |   .8713542   .3226843    -0.37   0.710     .4216708    1.800595
```

```
_Igruposani_3 |   1.107404   .6740677     0.17   0.867     .3358822    3.651115

        _cons |   1.471571    .582522     0.98   0.329     .6773806    3.196905

------------------------------------------------------------------------------


. xi:logistic I122 i.sexo i.edadgroup  i.gruposanitario i.Formacion

i.sexo            _Isexo_1-2          (naturally coded; _Isexo_1 omitted)

i.edadgroup       _Iedadgroup_1-3     (naturally coded; _Iedadgroup_1 omitted)

i.gruposanita~o   _Igruposani_1-3     (naturally coded; _Igruposani_1 omitted)

i.Formacion       _IFormacion_0-1     (naturally coded; _IFormacion_0 omitted)


Logistic regression                               Number of obs   =        286

                                                  LR chi2(6)      =       7.67

                                                  Prob > chi2     =     0.2634

Log likelihood = -190.69067                       Pseudo R2       =     0.0197


------------------------------------------------------------------------------

         I122 | Odds Ratio   Std. Err.      z    P>|z|     [95% Conf. Interval]

--------------+---------------------------------------------------------------

     _Isexo_2 |   .9781002   .2784276    -0.08   0.938     .5598593    1.708786

_Iedadgroup_2 |   .4439237     .18125    -1.99   0.047     .1994208    .9882031

_Iedadgroup_3 |   .4369264   .1558975    -2.32   0.020     .2171178    .8792678

_Igruposani_2 |   .7924303   .3013941    -0.61   0.541     .3760225    1.669968

_Igruposani_3 |   1.118351   .6808737     0.18   0.854     .3391178    3.688129

_IFormacion_1 |   3.535227   3.165222     1.41   0.158     .6113705    20.44232

        _cons |   1.479953   .5872142     0.99   0.323     .6800006    3.220971

------------------------------------------------------------------------------
```

Formar y condenar

```
. xi:logistic I123b i.sexo i.edadgroup  i.gruposanitario

i.sexo            _Isexo_1-2          (naturally coded; _Isexo_1 omitted)

i.edadgroup       _Iedadgroup_1-3     (naturally coded; _Iedadgroup_1 omitted)

i.gruposanita~o   _Igruposani_1-3     (naturally coded; _Igruposani_1 omitted)


Logistic regression                               Number of obs   =        286

                                                  LR chi2(5)      =       3.84

                                                  Prob > chi2     =     0.5727

Log likelihood =   -185.548                       Pseudo R2       =     0.0102
```

```
------------------------------------------------------------------------------
       I123b | Odds Ratio   Std. Err.      z    P>|z|     [95% Conf. Interval]
-------------+----------------------------------------------------------------
     _Isexo_2 |    .984321   .2853235    -0.05   0.957     .5576996    1.737293
_Iedadgroup_2 |   .4622812   .1910725    -1.87   0.062     .2056284    1.039272
_Iedadgroup_3 |   .5662726   .1997039    -1.61   0.107     .2836857    1.130352
_Igruposani_2 |    1.00759   .3783072     0.02   0.984      .482718    2.103167
_Igruposani_3 |   1.467955   .8971439     0.63   0.530     .4430975    4.863248
        _cons |   .9640544    .380971    -0.09   0.926     .4443521    2.091587
------------------------------------------------------------------------------

. xi:logistic I123b i.sexo i.edadgroup  i.gruposanitario i.Formacion
i.sexo            _Isexo_1-2          (naturally coded; _Isexo_1 omitted)
i.edadgroup       _Iedadgroup_1-3     (naturally coded; _Iedadgroup_1 omitted)
i.gruposanita~o   _Igruposani_1-3     (naturally coded; _Igruposani_1 omitted)
i.Formacion       _IFormacion_0-1     (naturally coded; _IFormacion_0 omitted)

Logistic regression                               Number of obs   =        286
                                                  LR chi2(6)      =       4.43
                                                  Prob > chi2     =     0.6187
Log likelihood = -185.25266                       Pseudo R2       =     0.0118

------------------------------------------------------------------------------
       I123b | Odds Ratio   Std. Err.      z    P>|z|     [95% Conf. Interval]
-------------+----------------------------------------------------------------
     _Isexo_2 |   .9885462   .2870435    -0.04   0.968     .5595439    1.746465
_Iedadgroup_2 |   .4619072    .190938    -1.87   0.062     .2054446     1.03852
_Iedadgroup_3 |   .5535589   .1960119    -1.67   0.095     .2765403    1.108075
_Igruposani_2 |    .959148   .3668405    -0.11   0.913     .4532427     2.02974
_Igruposani_3 |   1.476143   .9020695     0.64   0.524     .4456152    4.889864
_IFormacion_1 |   1.923364   1.625578     0.77   0.439     .3669847    10.08034
        _cons |   .9664228   .3820845    -0.09   0.931     .4452835     2.09748
------------------------------------------------------------------------------
```

Control

```
. xi:logistic I124 i.sexo i.edadgroup  i.gruposanitario
i.sexo            _Isexo_1-2          (naturally coded; _Isexo_1 omitted)
```

```
i.edadgroup       _Iedadgroup_1-3     (naturally coded; _Iedadgroup_1 omitted)
i.gruposanita~o   _Igruposani_1-3     (naturally coded; _Igruposani_1 omitted)

Logistic regression                               Number of obs   =        286
                                                  LR chi2(5)      =       6.46
                                                  Prob > chi2     =     0.2639
Log likelihood = -184.78902                       Pseudo R2       =     0.0172

------------------------------------------------------------------------------
        I124 | Odds Ratio   Std. Err.      z    P>|z|     [95% Conf. Interval]
-------------+----------------------------------------------------------------
     _Isexo_2 |   1.029073   .2988892     0.10   0.921     .5823965    1.818333
_Iedadgroup_2 |    2.87316   1.304051     2.33   0.020     1.180381    6.993548
_Iedadgroup_3 |   2.097421   .8616918     1.80   0.071     .9375252    4.692325
_Igruposani_2 |   1.279337   .4714773     0.67   0.504      .621283    2.634392
_Igruposani_3 |   1.063222    .649385     0.10   0.920     .3211699     3.51976
        _cons |    .266004   .1196862    -2.94   0.003     .1101279    .6425084
------------------------------------------------------------------------------

. xi:logistic I124 i.sexo i.edadgroup  i.gruposanitario i.Formacion
i.sexo            _Isexo_1-2          (naturally coded; _Isexo_1 omitted)
i.edadgroup       _Iedadgroup_1-3     (naturally coded; _Iedadgroup_1 omitted)
i.gruposanita~o   _Igruposani_1-3     (naturally coded; _Igruposani_1 omitted)
i.Formacion       _IFormacion_0-1     (naturally coded; _IFormacion_0 omitted)

Logistic regression                               Number of obs   =        286
                                                  LR chi2(6)      =       6.56
                                                  Prob > chi2     =     0.3639
Log likelihood = -184.74187                       Pseudo R2       =     0.0174

------------------------------------------------------------------------------
        I124 | Odds Ratio   Std. Err.      z    P>|z|     [95% Conf. Interval]
-------------+----------------------------------------------------------------
     _Isexo_2 |   1.027783   .2986078     0.09   0.925     .5815627    1.816377
_Iedadgroup_2 |   2.874565   1.304839     2.33   0.020     1.180837    6.997684
_Iedadgroup_3 |   2.116308   .8716441     1.82   0.069     .9440482    4.744204
_Igruposani_2 |   1.304274   .4876774     0.71   0.477     .6267558    2.714183
```

```
_Igruposani_3 |   1.061252   .6480936     0.10   0.922     .3206271    3.512666
_IFormacion_1 |   .7622814   .6821721    -0.30   0.762     .1319374    4.404158
        _cons |   .2655769    .119543    -2.95   0.003     .1099114    .6417087
------------------------------------------------------------------------------
```

1.2 DETECTAN CASOS DE RIESGO CORRECTAMENTE

```
. xi:logistic DetectaCRcorrecto i.sexo i.edadgroup  i.gruposanitario
i.sexo            _Isexo_1-2          (naturally coded; _Isexo_1 omitted)
i.edadgroup       _Iedadgroup_1-3     (naturally coded; _Iedadgroup_1 omitted)
i.gruposanita~o   _Igruposani_1-3     (naturally coded; _Igruposani_1 omitted)

Logistic regression                               Number of obs   =        286
                                                  LR chi2(5)      =       2.91
                                                  Prob > chi2     =     0.7139
Log likelihood =  -183.6529                       Pseudo R2       =     0.0079

----------------------------------------------------------------------------------
DetectaCRcorrecto | Odds Ratio   Std. Err.      z    P>|z|     [95% Conf. Interval]
------------------+---------------------------------------------------------------
         _Isexo_2 |   .9078295   .2637371    -0.33   0.739     .5137104    1.604317
    _Iedadgroup_2 |   .6825681   .2870507    -0.91   0.364     .2993487    1.556376
    _Iedadgroup_3 |   .8273206   .2984898    -0.53   0.599     .4079135    1.677952
    _Igruposani_2 |   1.628369   .5927453     1.34   0.180     .7978212    3.323534
    _Igruposani_3 |   1.591402   .9736292     0.76   0.448     .4797443    5.278981
            _cons |    .652033   .2621724    -1.06   0.288     .2964931    1.433919
----------------------------------------------------------------------------------

. xi:logistic DetectaCRcorrecto i.sexo i.edadgroup  i.gruposanitario i.Formacion
i.sexo            _Isexo_1-2          (naturally coded; _Isexo_1 omitted)
i.edadgroup       _Iedadgroup_1-3     (naturally coded; _Iedadgroup_1 omitted)
i.gruposanita~o   _Igruposani_1-3     (naturally coded; _Igruposani_1 omitted)
i.Formacion       _IFormacion_0-1     (naturally coded; _IFormacion_0 omitted)

Logistic regression                               Number of obs   =        286
                                                  LR chi2(6)      =       3.01
```

```
                                                  Prob > chi2     =     0.8076
Log likelihood = -183.60296                       Pseudo R2       =     0.0081

------------------------------------------------------------------------------------
DetectaCRcorrecto | Odds Ratio   Std. Err.      z    P>|z|     [95% Conf. Interval]
------------------+-----------------------------------------------------------------
         _Isexo_2 |   .9068212   .2635361    -0.34   0.736     .5130379    1.602854
    _Iedadgroup_2 |   .6826432   .2871479    -0.91   0.364     .2993252    1.556841
    _Iedadgroup_3 |   .8349761   .3022455    -0.50   0.618     .4107289    1.697434
    _Igruposani_2 |    1.66051   .6133603     1.37   0.170     .8050522    3.424987
    _Igruposani_3 |   1.588026   .9717085     0.76   0.450     .4786408    5.268723
    _IFormacion_1 |   .7555854    .678663    -0.31   0.755     .1299387    4.393681
            _cons |   .6509903   .2618647    -1.07   0.286     .2959195    1.432107
------------------------------------------------------------------------------------
```

1.3 CONOCEN PROTOCOLO DE ACTUACIÓN

```
. xi:logistic protocolo i.sexo i.edadgroup  i.gruposanitario
i.sexo            _Isexo_1-2          (naturally coded; _Isexo_1 omitted)
i.edadgroup       _Iedadgroup_1-3     (naturally coded; _Iedadgroup_1 omitted)
i.gruposanita~o   _Igruposani_1-3     (naturally coded; _Igruposani_1 omitted)

Logistic regression                               Number of obs   =        286
                                                  LR chi2(5)      =      36.50
                                                  Prob > chi2     =     0.0000
Log likelihood = -56.801976                       Pseudo R2       =     0.2431

--------------------------------------------------------------------------------
    protocolo | Odds Ratio   Std. Err.      z    P>|z|     [95% Conf. Interval]
--------------+-----------------------------------------------------------------
     _Isexo_2 |   1.061636   .6885566     0.09   0.927     .2977847    3.784849
_Iedadgroup_2 |   1.065632   .9013558     0.08   0.940     .2030604    5.592281
_Iedadgroup_3 |   .7931225   .6078105    -0.30   0.762     .1766122     3.56172
_Igruposani_2 |   21.96904   11.93388     5.69   0.000     7.575772    63.70821
_Igruposani_3 |    7.30798   6.619345     2.20   0.028      1.23824    43.13102
        _cons |   .0279603   .0260287    -3.84   0.000     .0045097    .1733563
```

```
------------------------------------------------------------------------------

. xi:logistic protocolo i.sexo i.edadgroup  i.gruposanitario i.Formacion
i.sexo           _Isexo_1-2          (naturally coded; _Isexo_1 omitted)
i.edadgroup      _Iedadgroup_1-3     (naturally coded; _Iedadgroup_1 omitted)
i.gruposanita~o  _Igruposani_1-3     (naturally coded; _Igruposani_1 omitted)
i.Formacion      _IFormacion_0-1     (naturally coded; _IFormacion_0 omitted)

Logistic regression                               Number of obs   =        286
                                                  LR chi2(6)      =      56.51
                                                  Prob > chi2     =     0.0000
Log likelihood = -46.795944                       Pseudo R2       =     0.3765

------------------------------------------------------------------------------
   protocolo | Odds Ratio   Std. Err.      z    P>|z|     [95% Conf. Interval]
-------------+----------------------------------------------------------------
     _Isexo_2 |   1.138465   .8402416     0.18   0.861     .2679736    4.836678
_Iedadgroup_2 |   1.003694   .8706428     0.00   0.997      .183331    5.494984
_Iedadgroup_3 |   .4451455   .3678913    -0.98   0.327     .0881099    2.248946
_Igruposani_2 |   26.38863   16.80467     5.14   0.000     7.574588    91.93369
_Igruposani_3 |   10.59454   10.24813     2.44   0.015     1.591153    70.54272
_IFormacion_1 |   195.6429   263.8339     3.91   0.000     13.91802    2750.113
        _cons |   .0236617   .0240983    -3.68   0.000     .0032147     .174162
------------------------------------------------------------------------------
```
